# Overcoming EMT-driven therapeutic resistance by BH3 mimetics

**DOI:** 10.18632/oncoscience.93

**Published:** 2014-11-05

**Authors:** Ulrike Keitel, Christina Scheel, Matthias Dobbelstein

**Affiliations:** ^1^ Institute of Molecular Oncology, Göttingen Center of Molecular Biosciences (GZMB), Faculty of Medicine, University of Göttingen, Göttingen, Germany; ^2^ Institute for Stem Cell Research, Helmholtz Center Munich, Neuherberg, Germany

**Keywords:** EMT, HMEC, HMLE

## Abstract

Epithelial-mesenchymal transition (EMT) contributes to the progression of cancer through enhanced invasion and stem-like properties of cancer cells. Additionally, EMT confers resistance towards many chemotherapeutics. We recently described a mechanism that mediates EMT-driven chemoresistance through augmented levels of Bcl-xL, an anti-apoptotic member of the Bcl-2 family (Keitel et al., Oncotarget, in press). Here, we elaborate on how these findings pertain to cancer cells dispersed in the tumor-adjacent stroma of breast cancer tissues, and how BH3-mimetics may provide a therapeutic strategy to eliminate cancer cell populations that have passed through an EMT.

Epithelial-mesenchymal transition (EMT) has long been known as a mechanism to enhance plasticity during development and wound healing. An extension of this model, however, proposes that EMT forms a key mechanistic basis for the progression of malignant tumors (reviewed by Nieto MA [1]). Most solid cancers are derived from epithelia, and such malignancies are referred to as carcinomas. Corresponding to the primary function of epithelial tissues, i. e. forming a barrier between compartments, epithelial cells form tight adhesions between each other, and display only lateral mobility, confined by the basement membrane. Initially, these features appear to be retained by carcinoma cells during tumor formation. However, a key hallmark of cancer progression entails invasion and metastasis [2]. Thus, epithelial tumor cells must undergo phenotypical changes that allow them to breach the basement membrane, invade adjacent tissues and ultimately form distant metastases. To accomplish these steps, at least parts of an EMT-like process appear to be necessary [3]. An instructive model of EMT is based on an experimental system employing normal primary human mammary epithelial cells (HMECs). These cells were immortalized by retroviral transduction of the SV40 T antigen and Telomerase reverse transcriptase (TERT), the catalytic subunit of the telomerase complex [4], and then termed HMLE (Human Mammary with Large T and TERT). Most of these cells are epithelial in phenotype. However, they contain a subpopulation of cells that display mesenchymal markers and morphology [5, 6]. This mesenchymal subpopulation (MSP) can be enriched by cell sorting, based on the surface markers CD44 (upregulated on mesenchymal cells) vs. CD24 (on epithelial cells). Importantly, MSP cells display enhanced mobility and invasiveness, thus recapitulating EMT-driven tumor cell invasion.

More recently, the process of EMT was associated with the ability of cells to initiate experimental tumors – a key-trait ascribed to “cancer stem cells”. Using an *in vitro* proxy assay, i.e. the ability of single cells to generate multicellular spheres in suspension (mammosphere assay), it was determined that the mesenchymal subpopulation of HMLE cells is enriched for mammosphere-forming cells. Moreover, following transformation with H-Ras, these cells initiated tumor xenografts in immunocompromised mice with higher efficiency than their epithelial counterparts [5]. Together, these results suggested that EMT contributes to the establishment of a small cell subpopulation in malignant tumors; this population is then capable of driving the regeneration of the tumor, even when the bulk tumor cell mass is destroyed by therapeutic regimens. As a corollary of this concept, to avoid relapse, anti-cancer therapies need to be tailored to include efficient targeting of this subpopulation.

Attempts to eliminate the tumor-initiating cell population generated by EMT are hampered by their increased resistance against most conventional cancer therapeutics. This is at least suggested by the observation that the mesenchymal subpopulation in the HMLE model remains viable upon most chemotherapeutic treatment regimens, under conditions that allowed the effective elimination of the epithelial population [7]. Therefore, to overcome chemoresistance mediated by EMT, drug screening efforts have focused on eliminating the mesenchymal subpopulation by searching for drugs with preferential cytotoxic effects on mesenchymal, rather than epithelial HMLE subpopulations [7]. While representing crucial conceptual advances in targeting strategies, such screens might be difficult to translate into the clinic, exemplified by the discovery of salinomycin, a drug that has been shown to preferentially kill mesenchymal breast cancer cells [7], but also displays high neurotoxicity [8]. Taken together, these observation impinge on one central problem: the development of rational targeting strategies aimed at overcoming therapeutic resistance require the precise elucidation of the molecular mechanisms whereby carcinoma cells that undergo EMT acquire the functional traits that render them resistant to conventional therapy.

According to this strategy, our group has defined molecular mechanisms that lead to the chemoresistance of mesenchymal tumor cells [9]. Firstly, we determined that the mesenchymal subpopulation of HMLEs was not only resistant towards various chemotherapeutics, but also to death receptors such as TNFalpha or Trail. This led us to suspect that anti-apoptotic gene products may lead to the general resistance of the mesenchymal cells. Indeed, several key players of the intrinsic apoptotic pathway were differentially regulated in their levels when comparing the epithelial and the mesenchymal subpopulation. Specifically, the inhibitor of apoptosis Bcl-xL (BCL2-like 1 isoform 1) was overexpressed in the mesenchymal cell population, and this turned out to be necessary and sufficient for chemoresistance. Moreover, the proapoptotic gene products BBC3/Puma and BCL2L11/Bim were downregulated in mesenchymal cells, possibly contributing further to their enhanced survival.

Importantly, the upregulation of Bcl-xL was not only observed in an experimental model system, but also in breast cancer patients. Thus, in a panel of breast cancer samples, we identified tumor cells that did not form part of the bulk tumor mass, but instead were interspersed within the adjacent stroma – as if forming the forefront of cancer cell invasion. Strikingly, this cancer cell subpopulation displayed enhanced expression of Bcl-xL, strongly suggesting that the upregulation of this antiapoptotic protein also supports the survival of invasive cancer cells within patients. Importantly, while the enhanced presence of Bcl-xL is expected to promote cancer cell survival in the first place, it may also indicate that the apoptotic machinery (especially the caspases) are still in place, making it necessary for the tumor cell to maintain a high level of Bcl-xL [10]. Thus, it is conceivable to expect at least some degree of tumor cell addiction to Bcl-xL, rendering it a desirable therapeutic target.

In an attempt to translate this molecular concept into a therapeutic approach, we applied BH3-mimetics. This class of drug candidates has gathered wide attention as a causal means to induce tumor cell apoptosis [11, 12]. It is based on mimicking the effects of proapoptotic proteins that only contain the BH3 domain that counteracts the activity of antiapoptotic regulators. Indeed, BH3 inhibitors were capable of eliminating not only the epithelial subpopulation of HMLEs, but also the mesenchymal HMLEs. This was true in vitro and, importantly, also in an animal model where H-Ras-transformed, mesenchymal HMLEs were used to generate xenograft tumors. Hence, BH3 mimetics might represent an attractive approach of eliminating resistant subpopulations of cancer cells, thereby targeting both tumor cell invasion and cancer relapse.

**Figure 1:**
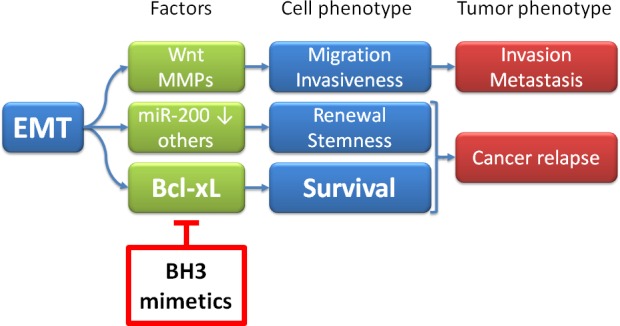
Antagonizing tumor progression by eliminating mesenchymal subpopulations Epithelial-mesenchymal transition (EMT) has three major consequences, displayed here in a simplified scheme: Invasion, tumor renewal (“stemness”), and survival. Factors involved in the three phenotypes are provided as examples, but this is by no means complete; they include the Wnt signaling pathway, enhanced synthesis of matrix metalloproteinases (MMPs), suppression of the family of microRNAs 200 (miR-200), and many others. A more comprehensive list of mechanisms that lead from EMT to invasiveness and/or stemness is provided in [13] and [14]. The contribution of Bcl-xL to the survival of cells that had undergone EMT is reported in a recent article in Oncotarget [9]. BH3 mimetics bear the potential of eliminating and otherwise resistant subpopulation of tumor cells that underwent EMT.
